# Assessing mechanisms of frequency discrimination by comparison of different measures over a wide frequency range

**DOI:** 10.1038/s41598-023-38600-0

**Published:** 2023-07-14

**Authors:** Brian C. J. Moore, Vinay

**Affiliations:** 1 Cambridge Hearing Group, Department of Psychology, University of Cambridge, Cambridge, UK; 2 Audiology Group, Department of Neuromedicine and Movement Science, Faculty of Medicine and Health Sciences, Norwegian University of Science and Technology (NTNU), Tungasletta 2, 7491 Trondheim, Norway

**Keywords:** Neuroscience, Psychology

## Abstract

It has been hypothesized that auditory detection of frequency modulation (FM) for low FM rates depends on the use of both temporal (phase locking) and place cues, depending on the carrier frequency, while detection of FM at high rates depends primarily on the use of place cues. To test this, FM detection for 2 and 20 Hz rates was measured over a wide frequency range, 1–10 kHz, including high frequencies for which temporal cues are assumed to be very weak. Performance was measured over the same frequency range for a task involving detection of changes in the temporal fine structure (TFS) of bandpass filtered complex tones, for which performance is assumed to depend primarily on the use of temporal cues. FM thresholds were better for the 2- than for the 20-Hz rate for center frequencies up to 4 kHz, while the reverse was true for higher center frequencies. For both FM rates, the thresholds, expressed as a proportion of the center frequency, were roughly constant for center frequencies from 6 to 10 Hz, consistent with the use of place cues. For the TFS task, thresholds worsened progressively with increasing frequency above 4 kHz, consistent with the weakening of temporal cues.

The auditory detection of changes in frequency has been proposed to depend on two mechanisms. The first, the “place” mechanism, depends on the frequency-to-place conversion that occurs in the cochlea; low frequencies produce maximum excitation towards the apex and high frequencies produce maximum excitation towards the base^1,2^. Changes in frequency may be coded by changes in the place of maximum response in the cochlea^1^, by changes on the apical side of the response pattern^3,4^, or by comparing changes in excitation level on the lower and upper sides of the excitation pattern^5–7^. Changes in frequency result in changes in excitation level that are largest on the low-frequency side of the excitation pattern, and these changes in excitation level are assumed to underlie frequency discrimination^3,8^. Place information is available over a very wide frequency range.

The second mechanism, the “temporal” mechanism, depends on the synchronisation of action potentials (spikes) in neurons of the auditory nerve to a specific phase of the waveform within the cochlea, which is called phase locking^9^. The time intervals between successive spikes are approximately integer multiples of the period of the input and this provides a potential code for frequency^10–12^. Phase locking in animals becomes less precise at high frequencies^9,13,14^. The same is probably true for humans, but the upper limit of phase locking in humans is not known. Some researchers have argued that phase locking plays a role in frequency discrimination for frequencies up to 8–10 kHz^11,12,15,16^, while some have argued that the limit is much lower, perhaps 1.5 kHz^17,18^.

One measure of frequency discrimination is the depth of frequency modulation (FM) required to distinguish a frequency modulated carrier from an unmodulated carrier^3,19,20^. This measure is denoted the FM detection limen (FMDL). It has been proposed that when the FM rate is below about 5 Hz, FMDLs depend partly on the use of temporal information when the carrier frequency is below about 5 kHz, while for FM rates above about 10 Hz, FMDLs depend largely on a place mechanism^6,21–24^. This is based on the idea that the mechanism that “decodes” the temporal information cannot track rapid changes in frequency^20,22^. However, the hypothesis that FM detection depends partly on temporal information for low FM rates and mainly on place information for high rates has been disputed^7,25^. A possible alternative explanation is described in the Discussion section.

Another measure of frequency discrimination, the difference limen for frequency (DLF), is the smallest detectable difference in frequency between successive steady tones^26^. The DLF has often been assessed using two tones with slightly different frequencies, presented in random order. The participant is asked to indicate whether the first or the second was higher in pitch. This task is difficult for untrained participants, who often find it hard to name the direction of a pitch change^27–29^. A task that requires less practice to achieve stable performance involves the use of four successive tones in each of two observation intervals. In one randomly selected interval the four tones have the same frequency, while in the other interval the tones alternate in frequency between two values. The participant is asked to identify the interval in which the tones changed in pitch^16,30^. Moore and Ernst^16^ used this task over a wide range of center frequencies. They found that DLFs, expressed as a proportion of center frequency, worsened with increasing frequency from 2 to 8 kHz and then became roughly constant with further increases in frequency. They suggested that the worsening in DLFs from 2 to 8 kHz reflected a progressive reduction in the precision of phase locking and that the “break point” around 8 kHz reflected a transition from a temporal code to a place code. However, this interpretation is controversial^18^.

Yet another measure of frequency discrimination involves the “TFS1” task^31–35^, which was specifically designed to limit the use of place cues. The participant is required to discriminate a harmonic complex tone (H), with fundamental frequency F0, from a similar tone in which all components are shifted up in frequency by ΔF (where ΔF ≤ 0.5F0), to create an inharmonic tone (I). The H and I tones have the same envelope repetition rate (equal to F0), but they differ in their temporal fine structure (TFS). The phases of the components are chosen randomly for each H and I tone, so the envelope shape fluctuates randomly from one tone to the next and does not provide a cue for discriminating the H and I tones. The H and I tones are made up of many components and are passed through a fixed bandpass filter centered on the higher unresolved components, to make place cues minimal. Therefore, it is assumed that performance of the TFS1 task involves the detection of changes in the TFS of the tones, conveyed by phase locking^36,37^. People with normal hearing perceive the H and I tones as having different pitches if ΔF is sufficiently large^38–40^. The task is similar to that used by Ernst and Moore^16^ to measure DLFs. One randomly selected interval contains the sequence HHHH and the other contains the sequence HIHI, and the participant is asked to identify the interval in which the tones changed in pitch.

The present study was designed to test some of the hypotheses described above by measuring FMDLs for 2 and 20 Hz rates and TFS1 thresholds over a very wide frequency range, including high frequencies, for which phase locking is assumed to be very weak. The following predictions were made:

1. For carrier frequencies for which phase locking is reasonably precise (up to about 4 kHz), FMDLs for the 2-Hz rate (denoted FM2) should be lower (better) than FMDLs for the 20-Hz rate (denoted FM20), because phase-locking cues are used for the former but not the latter.
2. For carrier frequencies where phase locking is very weak (above about 4 kHz), FM20 values should be lower than FM2 values, because the FM should be detected via the conversion of FM to amplitude modulation (AM) in the cochlea, and AM detection is better for a 20-Hz rate than for a 2-Hz rate^41–43^.
3. FM20 values, when expressed as a proportion of the carrier frequency, should be roughly invariant with frequency, because the slopes of excitation patterns and auditory filters are roughly invariant with frequency at medium to high frequencies^44^. This would be consistent with the results of Sęk and Moore^22^, although the highest FM rate that they used was 10 Hz and the highest carrier frequency that they used was 8 kHz.
4. Based on the assumption that the precision of phase locking decreases with increasing frequency, TFS1 thresholds should increase progressively with increasing center frequency above 4 kHz, and, unlike FM2 and FM20, should not flatten off for very high frequencies. This would be consistent with the results of Moore and Sęk^45^, who used center frequencies up to 4 kHz, and Moore and Sęk^46^, who used center frequencies of 8 and 10 kHz, although, to our knowledge, TFS1 thresholds have not been measured over a wide range of center frequencies using a single group of participants.

## Methods

### Participants

Participants were thirteen students at the Norwegian University of Science and Technology in Trondheim, Norway. They were recruited via flyers and via the university media channel. All had audiometric thresholds better than 20 dB HL (measured using an Otometrics Aurical Otosuite and Telephonics TDH-39P headphones) for all octave-spaced frequencies from 250 to 8000 Hz. Their ages ranged from 21 to 28 years. Nine were female and four were male. None of the participants had non-auditory neural conditions and none had any history of ear discharge, pain in their ears, or tinnitus. None of the participants had any neurological problems. Middle-ear function was checked using a Grason-Stadler Tympstar Pro tympanometer; all participants had normal middle-ear function. Participants were paid for participating by being given gift vouchers.

The study and methods followed the tenets of the Declaration of Helsinki, and informed consent was obtained from participants after the nature and possible consequences of participation were explained. Approval for the experiments was given by the Regional Committee for Medical and Health Research Ethics (REK) in Norway.

### Apparatus and test procedures

Stimuli were generated using the “PSYCHOACOUSTICS” software^47^ using the built-in 24 bit soundcard of a Microsoft Surface Pro laptop computer. Stimuli were delivered via Sennheiser HDA200 headphones, which have a smooth frequency response over a wide frequency range. Testing was carried out separately for each ear of each participant in a sound-attenuating room.

All thresholds were measured with a two-alternative forced-choice procedure using a two-down one-up adaptive procedure to estimate the 71% correct point on the psychometric function. The two observation intervals were marked on the laptop screen by successively lighting up two boxes. After the participant had responded, feedback was provided by flashing the correct box.

The following auditory measures were obtained.

1. Absolute thresholds for pure-tone signals for frequencies of 0.25, 0.5, 1, 2, 4, 6, 8, and 10 kHz. The signal duration was 1000 ms including 10-ms raised-cosine ramps. The two observation intervals were separated by 500 ms. The step size was 4 dB until four turnpoints had occurred and 2 dB thereafter. A run continued until 8 turnpoints (changes from decreasing to increasing level and vice versa) had been obtained. The threshold was taken as the arithmetic mean of the levels at the last six turnpoints. The threshold was measured twice for each ear, and the two estimates were averaged. For the measures of frequency discrimination, the level of the stimuli was set to be 50 dB SL, i.e., 50 dB above the absolute threshold.
2. FMDLs using modulation rates of 2 and 20 Hz and carrier frequencies of 1, 2, 4, 6, 8, and 10 kHz. The signal duration was 1000 ms including 20-ms raised-cosine ramps and the two intervals in a trial were separated by 300 ms. The carrier was unmodulated in one randomly chosen interval and frequency modulated in the other. The starting frequency deviation was 80 Hz, which was chosen to be well above the likely threshold value. The frequency deviation was changed by a factor of 1.95 until two turnpoints had occurred, then by a factor of 1.56 until two more turnpoints had occurred and then by a factor of 1.25. A run continued until 12 turnpoints had been obtained. The threshold was taken as the geometric mean of the frequency deviations at the last eight turnpoints. Two estimates of threshold were obtained for each ear, and the final threshold was taken as the geometric mean of the two. The thresholds are expressed as the peak-to-peak deviation from the carrier frequency.
3. TFS1 thresholds for center frequencies of 1, 2, 4, 6, 8, and 10 kHz. The stimuli were bandpass filtered complex tones. The tones were either harmonic (H) or all components were shifted up in frequency by ΔF, giving an inharmonic tone I. The H tone had an F0 of 0.11111 times the center frequency. For example, for the center frequency of 2 kHz the F0 was 222.22 Hz. The width of the bandpass filter was F0 and the lower edge of the passband fell at the nominal center frequency. Thus, the lowest component within the passband corresponded to the 9th harmonic. It was assumed that this would not be resolved in the auditory system^37,48^. To prevent the detection of combination tones, and to limit the audibility of components falling on the skirts of the bandpass filter, the tones were presented in a background of threshold equalising noise (TEN)^49^. The TEN started 300 ms before the first tone burst and ended 300 ms after the last tone burst. The TEN level is specified as the level in a 1-ERB_N_ wide band centered at 1000 Hz, where ERB_N_ stands for the average value of equivalent rectangular bandwidth of the auditory filter at moderate sound levels for listeners with normal hearing^44^. The level of the TEN was set 15 dB below the overall level of the complex tone. In one randomly selected interval of a trial, there were four successive 200-ms bursts (including 20-ms raised-cosine ramps) of tone H. The bursts were separated by 100 ms. In the other interval, tones H and I alternated, with the same 100-ms inter-burst interval, giving the pattern HIHI. The two intervals were separated by 300 ms. The participant was asked to choose the interval in which the sound changed across the four tone bursts within an interval. The phases of the components were chosen randomly for every tone burst, so the envelope of each tone burst was different and performance of the task could not be based on envelope cues. The starting value of ΔF was 0.5F0. This value of ΔF leads to the greatest possible difference between the H and I tones. The value of ΔF was changed by a factor of 1.95 until one turnpoint had occurred, then by a factor of 1.56 until one more turnpoint had occurred and then by a factor of 1.25. A run continued until eight turnpoints had been obtained. The threshold was taken as the geometric mean of the values of ΔF at the last six turnpoints. Two estimates of threshold were obtained for each ear, and the final threshold was taken as the geometric mean of the two.

For each measure of frequency discrimination, the nature of the task was carefully described to each participant, including what to “listen for”. When the initial performance of a participant was erratic or when a participant reported that they were not sure what to listen for, the participant was given practice and further instruction until their performance became stable. This happened only rarely, and only 1–2 practice runs were necessary for performance to become stable. The order of testing the FM detection thresholds and the TFS1 thresholds was randomised across participants. The ear that was tested first was varied randomly across participants.

## Results

The thresholds for each measure were generally similar for the two ears of each participant. To reduce the effects of random errors of measurement, the thresholds were averaged across ears (arithmetic means for absolute thresholds, geometric means for the measures of frequency discrimination). The measures of frequency discrimination were not correlated with the absolute thresholds for any center frequency (all *r* < 0.25, *p* > 0.41), which is not surprising, since all participants had absolute thresholds within the normal range.

**Figure 1:**
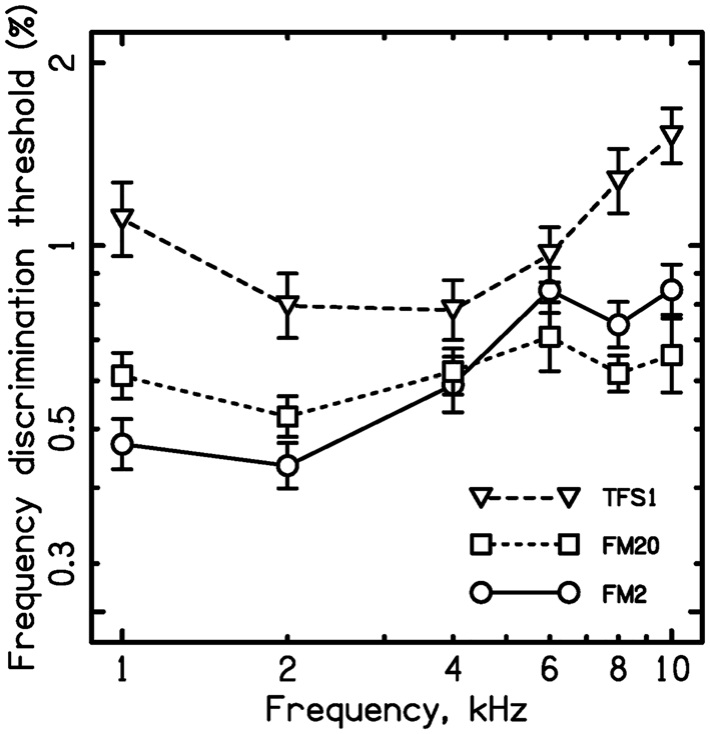
Geometric mean frequency discrimination thresholds expressed as a percentage of center frequency for the three measures: FM2, FM20, and TFS1. Error bars show ± 1 standard error.

For each measure and each center frequency, the thresholds were expressed as a percentage of the center frequency. The geometric mean thresholds across participants are shown in Fig. 1. A two-way ANOVA was conducted on the logarithms of the FM2, FM20 and TFS1 thresholds with factors carrier frequency and measure (FM2, FM20 and TFS1). In this and subsequent ANOVAs, the Huynh–Feldt correction was used when appropriate, but the uncorrected degrees of freedom are reported. Post hoc tests were conducted only when there was a corresponding significant main effect in the ANOVA, in which case Fisher’s LSD tests were used, uncorrected for multiple comparisons^50^. There were significant main effects of carrier frequency [*F*(5, 60) = 10.5, *p* < 0.001] and of measure: *F*(2,24) = 35.9, *p* =  < 0.001. The main effect of measure occurred because TFS1 thresholds were higher overall than FM2 thresholds and FM20 thresholds (both *p* < 0.001). This may have been partly a consequence of the fact that the TFS1 stimuli were presented in a background TEN, while the FM2 and FM20 thresholds were determined in quiet. Noise would be expected to disrupt the internal representation of both temporal cues and place cues. Importantly, there was a significant interaction of carrier frequency and measure [*F*(10, 120) = 6.82, *p* < 0.001], confirming that the pattern of thresholds across frequency differed across measures.

For carrier frequencies from 6 to 10 kHz, FM2 values were higher than FM20 values, while for the carrier frequencies of 1 and 2 kHz the opposite was true. A two-way ANOVA on the logarithms of the FM2 and FM20 thresholds with factors carrier frequency and FM rate showed that the main effect of frequency was significant [*F*(5, 60) = 10.1, *p* < 0.001] but the main effect of FM rate was not significant: *F*(1,12) = 0.42, *p* = 0.53. Importantly, there was a significant interaction of frequency and FM rate: *F*(5, 60) = 6.30, *p* < 0.001. Post hoc LSD tests showed that at 1 kHz FM2 thresholds were significantly lower than FM2 thresholds (*p* = 0.018), while at 8 kHz FM2 thresholds were significantly higher than FM2 thresholds (*p* = 0.004). The finding that FM2 thresholds were lower than FM20 thresholds for low carrier frequencies is consistent with the hypothesis that FM2 thresholds were partly based on the use of temporal information. The finding that FM2 thresholds were higher than FM20 thresholds at high carrier frequencies is consistent with the hypothesis that for high carrier frequencies FM detection for both rates was determined largely via FM to AM conversion. The magnitude of the AM cues would have been the same for the two FM rates, but AM detection for a 20-Hz rate is better than AM detection for a 2-Hz rate^41–43^, leading to better FM detection for the higher rate.

The FM20 thresholds varied only slightly with center frequency. A one-way repeated-measures analysis of variance (ANOVA) was conducted on the logarithms of the FM20 values with carrier frequency as the factor. The effect of carrier frequency was not significant: *F*(5, 60) = 2.45, *p* = 0.081. This is consistent with the hypothesis that FM20 values were largely based on place cues, i.e. on FM to AM conversion, and that the salience of these cues did not vary markedly with carrier frequency.

The FM2 thresholds increased with increasing carrier frequency from 2 to 6 kHz and then remained roughly constant. A one-way ANOVA conducted on the logarithms of the FM2 thresholds showed that the effect of carrier frequency was significant: *F*(5, 60) = 11.84, *p* < 0.001. Post hoc tests showed that FM2 thresholds at 4, 6, and 8 kHz were significantly higher than FM2 thresholds at 1 and 2 kHz (all *p* < 0.003), but FM2 thresholds did not differ significantly for the carrier frequencies of 6, 8, and 10 kHz (*p* > 0.305). This pattern of results is consistent with the hypothesis that FM2 values depended partly on the use of temporal cues for low and medium frequencies, that the availability of these cues decreased with increasing carrier frequency above 2 kHz, and that the temporal cues became largely unusable at 6 kHz, leading to roughly constant FM20 values for carriers from 6 to 10 kHz.

Unlike the FM2 and FM20 thresholds, the TFS1 thresholds increased progressively with increasing carrier frequency from 4 to 10 kHz. A one-way ANOVA conducted on the logarithms of the TFS1 values showed that the effect of carrier frequency was significant: *F*(5, 60) = 8.97, *p* < 0.001. Post hoc LSD tests showed that TFS1 thresholds were significantly higher for the center frequency of 10 kHz than for the center frequencies of 8 kHz (*p* = 0.026), 6 kHz (*p* < 0.001) and 4 kHz (*p* < 0.001). Also TFS1 thresholds were significantly higher for the center frequency of 8 kHz than for the center frequencies of 4 kHz (*p* = 0.006) and 2 kHz (*p* = 0.023). The TFS thresholds increased significantly when the frequency was reduced from 2 to 1 kHz, and this difference was significant (*p* = 0.031). A similar effect was observed by Moore and Sek^45^. The effect may reflect the fact that there were more TFS and envelope peaks in the stimulus at 2 than at 1 kHz. Alternatively, or in addition, the auditory system may be less accurate in using inter-spike intervals when the relevant intervals are long, as they are for low F0s^51^.

## Discussion

The variation of the FM2 thresholds with carrier frequency was similar to that found by Sęk and Moore^22^, although they tested only three participants and the highest carrier frequency that they used was 8 kHz. Sęk and Moore did not measure FM detection thresholds for a rate of 20 Hz, but their results for a rate of 10 Hz are similar to those found here for a rate of 20 Hz, showing little variation with carrier frequency over the range 1–8 kHz in their data or 1–10 kHz in our data. The pattern of results for FM2 and FM20 is also similar to that found by Whiteford and Oxenham^52^ for sinusoidal carriers presented in TEN, although they found that FM20 thresholds decreased slightly when the carrier frequency was increased from 1.4 to 4 kHz. The small variation of FM2 thresholds across medium to high carrier frequencies is consistent with the idea that, for FM rates of 10 Hz and above, FM detection depends largely on FM-to-AM conversion. The ability to use such cues depends on the sharpness of tuning in the cochlea and the ability to detect fluctuations in excitation level at a given place in the cochlea. The sharpness of the excitation pattern evoked by a frequency-modulated carrier does not depend on FM rate provided that the spectral components of the stimulus are not resolved, but, as noted earlier, the ability to detect AM for a stimulus of fixed duration is markedly better for a 20-Hz rate than for a 2-Hz rate^41–43^, probably because there are more modulation cycles in the stimulus at the higher rate. Hence, over the frequency range where temporal cues are very weak, the FM20 values should be markedly smaller than the FM2 values. The results were consistent with the expected pattern: FM20 values were smaller than FM2 values for the carrier frequencies of 6, 8, and 10 kHz. The opposite pattern was found for the carrier frequencies of 1 and 2 kHz, consistent with the idea that over the frequency range where salient temporal cues are available, FM detection at low rates is based partly on the use of temporal cues.

A possible alternative explanation for the pattern of results for FM2 and FM20 is provided by the results of Whiteford et al.^7^. In principle, FM detection might depend on comparison of the relative phase of the excitation level fluctuations on the two sides of the excitation pattern. A similar cue might be used to distinguish AM from FM^6^. For FM, the fluctuations are 180° out of phase, while for AM they are in phase. Whiteford et al. showed that sensitivity to the relative phase of AM applied to two closely-spaced carriers (which is similar to comparing the relative phase of the excitation level fluctuations on the two sides of the excitation pattern) followed the same trends with carrier frequency and AM rate as FM detection. They concluded that “The results suggest a unitary place-based neural code for FM across all rates and carrier frequencies”. However, the results of a similar experiment conducted by Moore and Sęk^6^ are difficult to explain in terms of the account of Whiteford et al.^7^. Moore and and Sęk found that performance in discriminating in-phase from out-of-phase AM applied to two closely spaced carriers (762 and 1296 Hz, presented together with a narrowband noise centered at 1000 Hz to mask cues resulting from the interaction of the two carriers) was almost independent of AM rate for rates from 2 to 10 Hz, whereas FM detection thresholds for a 1000-Hz carrier increase with increasing rate over the same range^22^. Moore and and Sęk^6^ also found that, for an AM rate of 2 Hz, participants could not discriminate in-phase from 180° out-of-phase AM (performance was at chance) for low AM depths, whereas at similar modulation depths participants performed well in distinguishing AM from FM. Moore and Sęk concluded that the good AM-FM discrimination found for the 2-Hz modulation rate could not be explained in terms of comparison of the phase of AM on the two sides of the excitation pattern.

In another study, Whiteford and Oxenham^52^ measured FM detection for complex tones presented in TEN whose resolved harmonics all fell above 8 kHz, such that phase locking to the harmonics would have been very weak. Even for such tones, the sensitivity to FM was better for a 2-Hz than for a 20-Hz FM rate. They suggested that the pattern of FM detection thresholds as a function of carrier frequency and FM rate reflected the use of place-based cues for all FM rates and carrier frequencies and that performance was limited by a central mechanism. While it is hard to rule out this interpretation, it does not account for why FM2 thresholds increased with increasing carrier frequency from 2 to 6 kHz and then remained roughly constant.

The TFS1 thresholds increased with increasing center frequency above 4 kHz. This is consistent with the results of Moore and Sęk^45^, who used center frequencies up to 4 kHz, and Moore and Sęk^46,53^, who used center frequencies of 8 and 10 kHz. Sęk^46^ found that six out of eight of their participants could not complete the adaptive procedure for the center frequency of 8 kHz and four out of eight could not complete the procedure at 10 kHz. In contrast all of our participants were able to complete the adaptive procedure at both 8 and 10 kHz, although the thresholds for some of them approached the highest possible value. The H and I stimuli used in the TFS1 task differ most when the frequency shift is one-half of the nominal F0. For the center frequency of 10 kHz, the F0 was 1111.1 Hz, so the largest possible frequency shift was 555.5 Hz, corresponding to 5.56% of the center frequency. The discrepancy across studies may have occurred because Sęk and Moore^46^ used a passband width of 5F0 whereas here a passband width of F0 was used. The smaller passband width makes the TFS1 task somewhat easier, probably because it reduces the ambiguity of cues in the TFS of the stimuli^36^. The present results for the TFS1 task suggest that, when place cues are minimal, weak temporal cues can be used for frequency discrimination for center frequencies up to about 8–10 kHz, consistent with the results of Moore and Ernst^16^ and with some modelling studies^11,15^.

## Conclusions

These experiments were designed to test the following hypotheses:

1. Auditory detection of frequency modulation (FM) for low FM rates depends on the use of both temporal (phase locking) and place cues, depending on the carrier frequency; temporal cues dominate for low carrier frequencies and place cues dominate for high carrier frequencies.
2. Detection of FM at high rates depends primarily on the use of place cues over a wide frequency range.
3. In a task designed to minimize the availability of place cues and promote the use of temporal cues, the TFS1 task (which involves detection of changes in the TFS of bandpass filtered complex tones), performance should worsen markedly at high center frequencies because of the reduced availability of temporal cues.

To test these hypotheses, FM detection for 2 and 20 Hz rates was measured over a wide frequency range, 1–10 kHz, including high frequencies, for which temporal cues are assumed to be very weak. Performance was measured over the same frequency range for the TFS1 task. FM thresholds were better for the 2- than for the 20-Hz rate for center frequencies up to 4 kHz, while the reverse was true for higher center frequencies. For both FM rates, the thresholds, expressed as a proportion of the center frequency, were roughly constant for center frequencies from 6 to 10 Hz, consistent with the use of place cues. These results are consistent with hypotheses 1 and 2. For the TFS task, thresholds worsened progressively with increasing frequency above 4 kHz, consistent with the weakening of temporal cues at very high frequencies and with hypothesis 3. The results for the TFS1 task suggest that weak phase locking cues are available for center frequencies up to 8–10 kHz.

## Data Availability

The data for the current study are available from the corresponding author on reasonable request.
